# Premature aging of red wines: a matter of fatty acid oxidation? A review on lipid‐derived molecular markers and their possible precursors

**DOI:** 10.1002/jsfa.14382

**Published:** 2025-05-22

**Authors:** Ilaria Prezioso, Enrico Ottaviano, Giuseppe Corcione, Chiara Digiorgio, Gabriele Fioschi, Vito Michele Paradiso

**Affiliations:** ^1^ University of Salento Department of Biological and Environmental Sciences and Technologies Lecce Italy

**Keywords:** climate change, lipid oxidation, dried fruit aroma, PremOx, lactones, lipoxygenase, 3‐methyl‐2,4‐nonanedione

## Abstract

Premature aging of red wines (PremOx) is an emerging defect in red wines, related to the onset of cooked fruit, plum and dried figs nuances, as well as the loss of aging potential. This review is focussed on the contribute of lipid‐derived compounds, mainly originating from fatty acids oxidation, to this defect. Lactones [γ‐nonalactone, massoia lactone), ketones (3‐methyl‐2,4‐nonanedione, (*Z*)‐1,5‐octadien‐3‐one] and aldehydes [(2*E*,4*E*,6*Z*)‐nonatrienal and *trans*‐4,5‐epoxy‐(*E*)‐2‐decenal] are reported among the most relevant odorants involved. Their occurrence and sensory significance are discussed and their candidate precursors, as well as possible pathways of formation, involving either enzymatic or radical oxidation, are reported. The literature reports about the effect of over‐ripening, light exposure, disease infections, harvesting date and wine aging conditions are also discussed. Fatty acid oxidation could be the common origin of some of the compounds related to premature aging. Applying the principles of lipid chemistry to better understand the mechanisms underlying their formation and evolution could provide useful knowledge to adapt vineyard management, harvest and winemaking techniques to the emerging issues related to climate change. © 2025 The Author(s). *Journal of the Science of Food and Agriculture* published by John Wiley & Sons Ltd on behalf of Society of Chemical Industry.

## INTRODUCTION

Premature aging is a defect consisting in a rapid loss of the fruity aromas characteristic of young wines, with the appearance of an oxidized bouquet, without the pleasant ageing notes.^1^, ^2^, ^3^ This olfactory deviation can appear in all types of wine, regardless the grape variety and the winemaking technique. Premature aging in red wines, sometimes indicated as ‘*PremOx*’, appears with the onset of notes of dried fruits, prune and figs, overwhelming other aroma descriptors, and is showing an increasing occurrence in the context of climate change.^2^, ^3^, ^4^ Several compounds have been reported to contribute to these nuances^2^, ^5^ and several pathways seem to concur to their formation, including the Maillard reaction, hydrolysis of glycosidic precursors and fatty acid oxidation.^2^, ^6^ In particular, lipid oxidation involves a multitude of substrates, reactions and products.^7^, ^8^, ^9^, ^10^ Compared to other oxidation phenomena occurring in musts and wines and involving other substrates (e.g. phenolic compounds, alcohols, amino acids and sugars), lipid oxidation has its own chemistry, rules and pathways. Even though enzymatic oxidation (via the lipoxygenase pathway) is well known as a contributor of wine volatiles (i.e. C‐6 carbonyls and alcohols also known as green leaf volatiles),^11^, ^12^ the current knowledge about the contribution of lipid oxidation to the volatile profile of wine is currently fragmented and lacks a comprehensive approach involving autoxidation, photooxidation and other radical‐mediated phenomena, despite some pioneering studies on grape and wine lipidomics.^13^, ^14^, ^15^, ^16^, ^17^, ^18^, ^19^ However, attention has been often paid to wine lipids with other perspectives, such as for profiling scopes^14^, ^16^; in relation of fermentation performances^20^; in view of their biological activity,^21^, ^22^ as a means to obtain wine traceability;^23^ or to monitor yeast autolysis.^24^ Scarce contributions provide insight on the role of fatty acids as precursors and on the autoxidation pathways leading to the formation of sensorially relevant volatile compounds. We consider that a comprehensive lipidomic approach to the grape‐must‐wine system could provide new concepts and tools to understand and manage important phenomena contributing to the sensory profile of wines and to adapt viticulture and enology to the changing climatic scenarios.

In this perspective, this review aimed to collect evidence from the literature on the role of lipid‐derived compounds in the olfactory deviation of premature aging of red wines, their sensory impact and their relations with climate, as well as vineyard and grape management. Attention is also paid to their candidate fatty acid precursors, aiming to provide a basis for further research on lipidomics applied to the grape‐must‐wine system, in view of a comprehensive knowledge and of the development of management strategies of fatty acid oxidation and its impact on wine quality.

## LITERATURE SEARCH AND ANALYSIS

### Literature review guidelines

Even though this review was not conceived as a systematic review, the literature review regarding premature aging in red wine was carried out with the support of the PRISMA guidelines,^25^ with special reference to the PRISMA‐Search checklist,^26^ and as described in the following sections.

The research question guiding this study was: ‘is the onset of premature aging and of cooked fruits aroma in red wine related to volatile compounds deriving from fatty acid oxidation?’

### Information sources and methods

The literature search was carried out on Scopus and Web of Science databases. After record selection was carried out as described in the (Search strategies and Peer review), cited references and citing references were examined for each selected record. Citing references were examined on Scopus.

### Search strategies

The following entries were used for literature search: red AND wine AND premature AND aging; red AND wine AND premature AND oxidation; red AND wine AND premox; red AND wine AND cooked AND fruit. No limits or filters were applied to the search. The search was updated periodically during the writing and the reviewing processes. The last update was on 24 April 2025.

### Peer review

The literature search and the record management were carried out and checked by different coauthors.

### Managing records

In total, 135 records was obtained by the search on Scopus and Web of Science databases. After manual deduplication, a list of 86 records was obtained. A further screening step was carried out, leading to the exclusion of 44 records based on title and abstract contents that were considered irrelevant to the aim of this review. In particular, studies regarding human health and biology (*n* = 29), food matrices other than grape wine (12) and consumer science (*n* = 3) were excluded. The remaining records were examined via full text analysis. Studies not regarding premature aging, as well as studies not related to lipid‐derived volatile compounds were excluded (*n* = 30). The analysis of cited literature and citing literature of the resulting records lead to a final list of 23 research papers.

**Figure 1:**
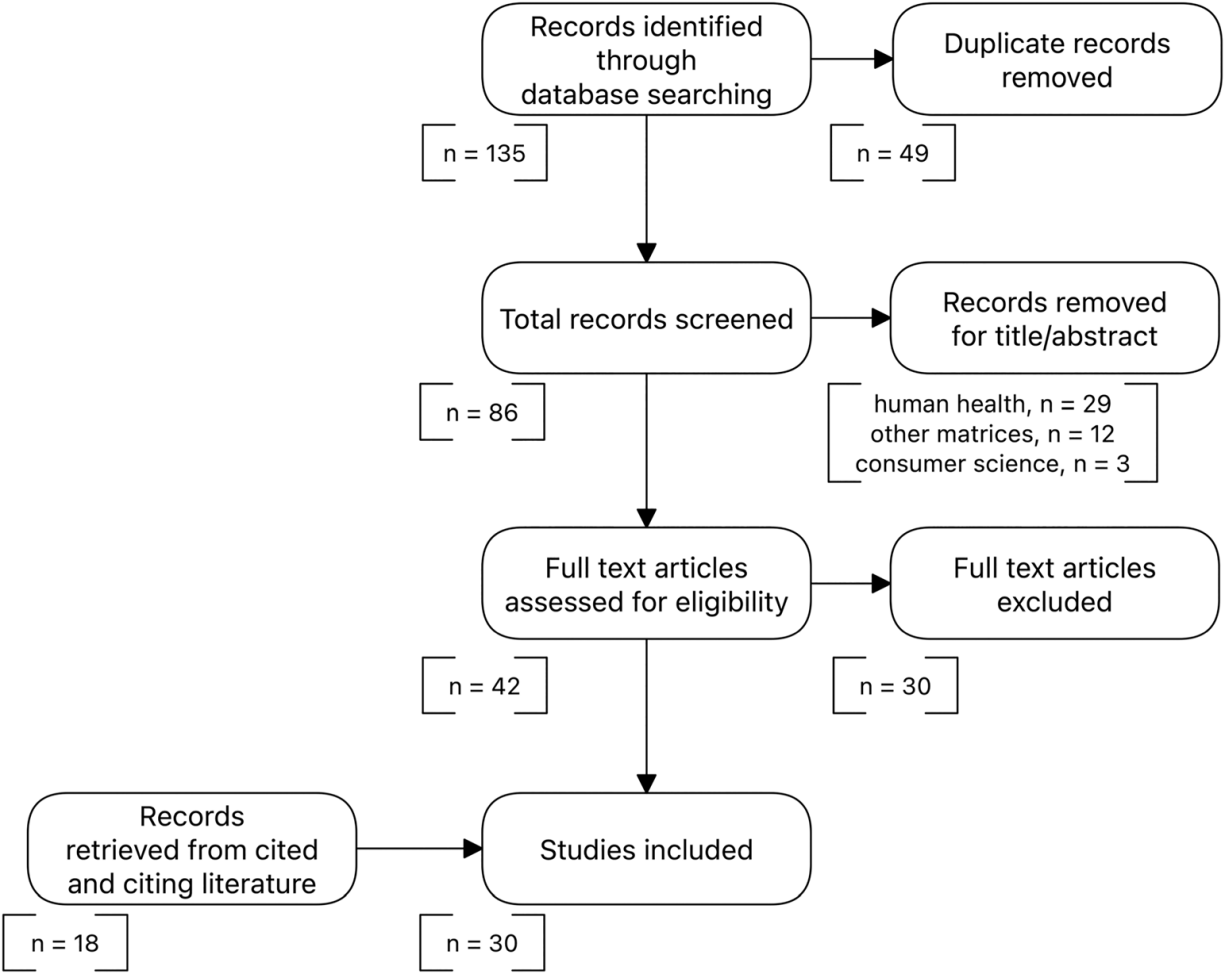
Scheme of the literature search procedure adopted for this review.

The described search procedure is schematized in Fig. 1.

## 3‐METHYL‐2,4‐NONANEDIONE: A KEY FOOD ODORANT

As regards red wines, obtained with a phase of contact between must and skins, premature ageing appears with unpleasant notes of cooked fruit, dried fruit and plum that prematurely replace the fresh fruity notes.

**Figure 2:**
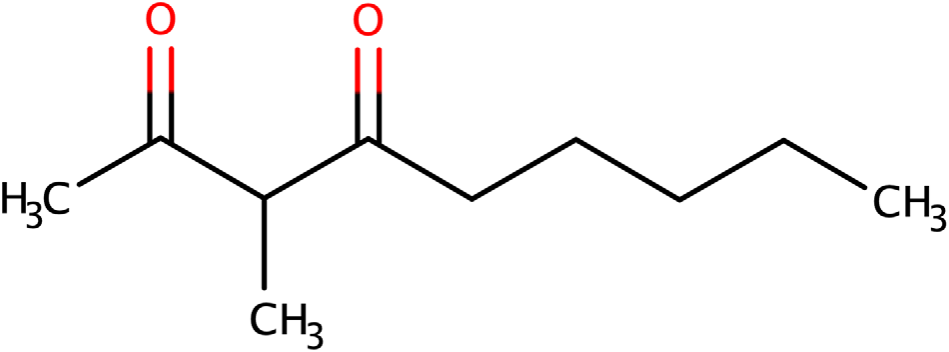
Structure of 3‐methly‐2,4‐nonanedione.

Gas chromatography‐olfactometry (GC‐O) was applied to investigate on the molecules responsible of prematurely ageing in red wines.^2^ The three ‘odoriferous zones’ (OZ) perceived by GC‐O as strongly associated with the prematurely aged aroma of red wine are listed in Table 1. OZ1 had a strong plum smell. OZ3, in addition to intensifying the plum smell, had an aroma similar to ripe peach. Therefore, only OZ1 and OZ3 appeared to be specific to prematurely aged red wines. Instead, OZ2 was detected in all wines analyzed, regardless of whether they had or had not prematurely aged character. Using GC‐mass spectrometry, it was possible to identify molecules corresponding to the following ‘odoriferous zone’: OZ1, 3‐methyl‐2,4‐nonanedione (MND); OZ2, β‐damascenone; and OZ3, γ‐nonalactone. These results therefore allowed an understanding of how the characteristic plum smell of prematurely aged red wines could be the result of a combination of several molecules including MND, β‐damascenone and γ‐nonalactone.^2^ The presence of the β‐diketone MND (Fig. 2)^2^, ^27^, ^28^ in red wines can be attributed to oxidative mechanisms, which involve the formation of oxygenated radicals during the winemaking process, ageing and storage. Indeed, the red wine's flavour undergoes evolution under oxidative conditions that can be summed up in four steps.^2^, ^27^ The first is the loss of fresh fruity flavours, followed by the development of aromatic nuances reminiscent plum and fig. These two steps constitute what is known as premature aging or ‘*PremOx*’. Rancid and turpentine flavours characterize the third and fourth phases, typically associated with the end of commercial life of aged red wines.^27^ Pons *et al*.^27^ thoroughly analyzed the role of MND in the flavour of aged red wines. They found a low perception threshold in red wine (16 ng L^−1^ and 62 ng L^−1^ in model wine and red wine, respectively) and that the presence of MND alone could significantly modify the flavour of the red wine. The preeminent descriptors associated with MND were related to its concentration (Table 2). Indeed, MND evokes flavours such as mint, hazelnut or anise, depending on its concentration. Adding MND to a fresh fruity red wine removed the ‘fresh fruit’ nuances resulting in the appearance of nuances ranging from fresh fruit flavour in red wine without MND to aromatic expression of *rancio* in wines with elevated concentrations (308.9 ng L^−1^), passing through mint/anise, plum and fig nuances. Therefore, it is possible to assert how MND content profoundly modifies the flavour of the wine based on its concentration.

**Table 1:** Identification of the ‘odoriferous zone’ responsible of the perception of prematurely aged aromas in red wine extracts

Aromatic profiles	Odour descriptors^a^	Identified compounds^b^
OZ1	Plum	3‐methyl‐2,4‐nonandione
OZ2	Dried fruit, applesauce	β‐damascenone
OZ3	Overripe peach	γ‐nonalactone

*Note*: Adapted with permission from Pons *et al*.^2^ Copyright © 2008 American Chemical Society.

^a^ Odour descriptors generated by two assessors during GC‐O.

^b^ Compounds identified as odour‐active, responsible of the perceived odour.

**Table 2:** Aromatic descriptors of 3‐methyl‐2,4‐nonandione based on the concentration detected in wine

Aromatic descriptors	Corresponding concentrations of MND (ng L‐1)
Fruity aromas	0
Mint, anise	90–170
Plum	170–250
Fig	250
Rancio	330

*Note*: Adapted with permission from Pons *et al*.^27^ Copyright © 2013 American Chemical Society.

The odorant receptors (ORs) OR1A1 and OR2W1, resident in nose's olfactory sensory neurons cilia,^29^, ^30^ have been identified as capable of recognize various odoriferous molecules,^31^, ^32^, ^33^, ^34^, ^35^ in particular *key food odorants* (KFO). MND has been reported as the most potent KFO for the OR1A1 receptor by three orders of magnitude among 190 KFOs.^36^ A study carried out by Geithe *et al*.^36^ revealed that OR1A1 was activated by MND in a wide concentration range (0.003–1500 μmol L^−1^).

Specifically, OR1A1 not only responded better to MND as a result of the presence of different conformations and/or binding sites located in the receptor capable of perceiving the compound at more concentrations, but also responded to γ‐nonalactone, thus underlining its role as an important receptor that participates in the identification of the plum and dried fruit characters usually found in oxidized wines.^36^

### MND precursors and related compounds

**Figure 3:**
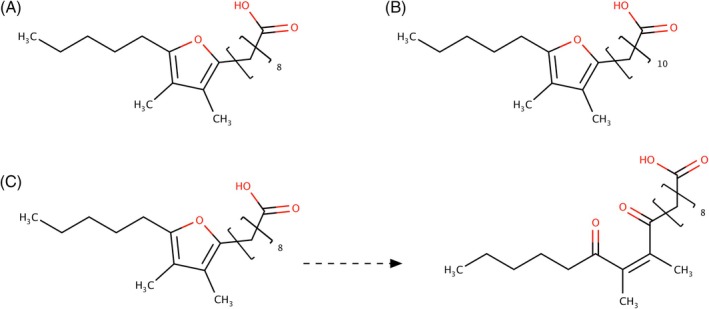
Structure of 10,13‐epoxy‐11,12‐dimethyloctadeca‐10,12‐dienoic acid (A) and 12,15‐epoxy‐13,14‐dimethyleicosa‐12,14‐dienoic acid (B). (C) Oxidation of FuFAs to dioxonenes (C).

MND was first mentioned by Guth and Grosch^37^ as an *off‐flavour* in inverted soybean oil. It was then associated with the unpleasant hay‐like flavour that develops in dried parsley^38^ and spinach.^39^ This β‐diketone has also been identified in green tea, enhancing its taste.^40^ Guth and Grosch^37^ discovered that some furan fatty acids (FuFAs: 10,13‐epoxy‐11,12‐dimethyloctadeca‐10,12‐dienoic acid and 12,15‐epoxy‐13,14‐dimethyleicosa‐12,14‐dienoic acid) (Fig. 3A,B) are precursors of this compound in soybean oil.

FuFAs are natural lipids, characterized by a furan ring which presents a linear fatty acid with 9, 11 or 13 carbon atoms in α position and an alkyl chain in the α' position. In the furan ring, one or both the β positions are replaced with a methyl group.^41^ Further studies have shown that these acids are found in plant tissues.^42^

FuFAs act as free radical scavengers, being oxidized via radical pathways to dioxoenes (Fig. 3C).^43^ This suggests their defence role against oxidative stress^43^, ^44^: indeed, they are mainly located in phospholipids where polyunsaturated fatty acids need to be protected. Therefore, the accumulation of furan fatty acids can be considered as one of the mechanisms through which plants defend themselves from oxidative stress.^45^ The formation of MND by photooxidation of FuFAs was confirmed^46^, ^47^ and MND was proposed as responsible of the light‐induced off‐flavour.

The presence of FuFAs in grapes can be attributed mainly to peel and seeds because they both contain fatty acids. Fruit development and ripening are involved in oxidation mechanisms as a result of the accumulation of reactive oxygen species (ROS), from the beginning of ripening to mature stage,^48^ and especially during over‐ripening.^49^ Muñoz & Munné‐Bosch^49^ highlighted the mechanisms of photooxidation and plant response during fruit ripening. This could be the frame of FuFAs accumulation and MND formation as an effect of their photooxidation.

Because red winemaking techniques allow contact between liquid and solid grapes fraction, a release of these acids could occur during maceration operations, leading to the formation of MND in red wines. The role of the lipid fraction of grape solids in the genesis of MND was also confirmed by an investigation on cognacs and spirits^50^ that reported higher levels of MND in Grappas, obtained by distillation of lees, and a strong correlation between the content of MND in distillates and the content of ethyl esters of fatty acids. On the other hand, non‐oxidized white wines, obtained without contact with grape solids, are characterized by the lowest levels of MND.^27^

**Figure 4:**
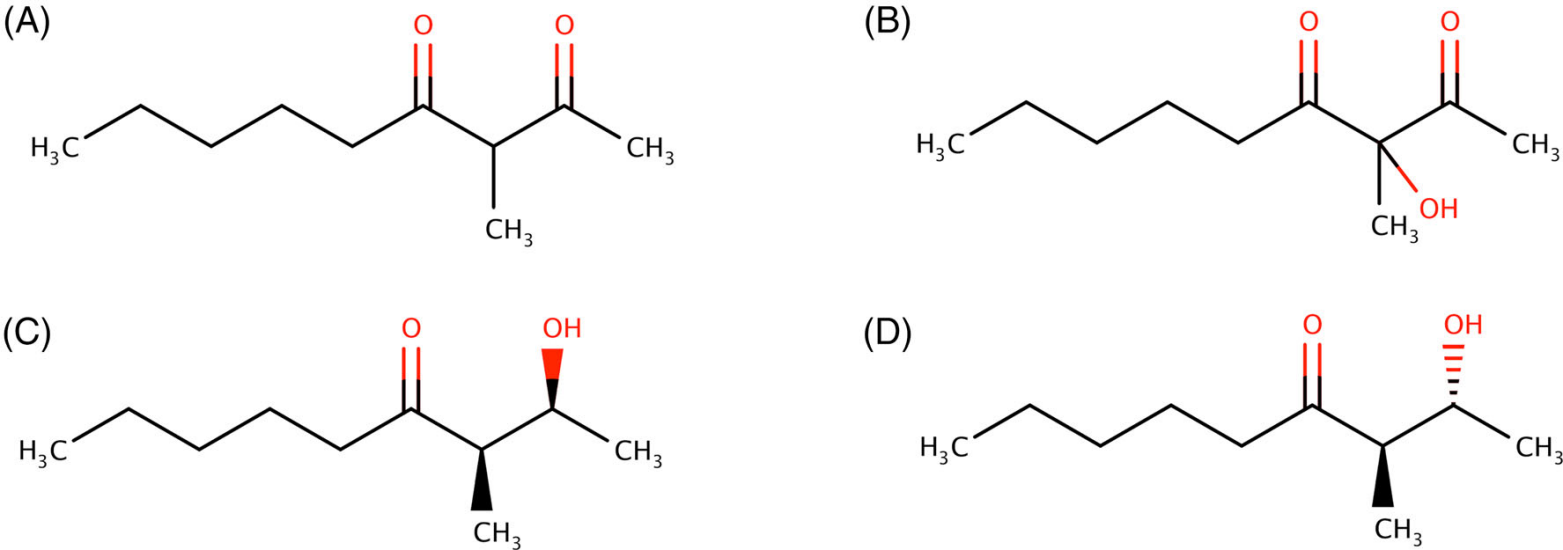
Structures of MND (A), 3‐hydroxy‐3‐methyl‐2,4‐nonanedione (B), 2‐hydroxy‐3‐methylnonan‐4‐one, and *syn*‐ (C) and *anti*‐ketol (D) diastereoisomers.

Another way of formation of MND in wine has been suggested by Peterson *et al*.,^51^ who identified two new hydroxy‐ketones, 2‐hydroxy‐3‐methylnonan‐4‐one (*syn*‐ and *anti*‐ketol diastereoisomers) and 3‐hydroxy‐3‐methyl‐2,4‐nonanedione (HMND) in musts and wines (Fig. 4). Peterson *et al*.^51^ reported a decrease of MND and HMND during alcoholic fermentation, attributed to a β‐ketoreductase in *Saccharomyces cerevisiae* able to transform MND by reducing the carbonyl group to a hydroxyl group producing a diastereomeric mixture of ketols.^5^ These compounds would act as precursors that could be spontaneously re‐oxidized to MND during wine aging under oxidative conditions.^51^

Moreover, Sigrist *et al*.^52^ reported that HMND could also originate from MND by photooxidation. Its sensory impact could be detrimental because it has been described as earth, plastic and rubbery.

### MND distribution in wines

It has been reported that the amount of MND changes considerably in different types of wine from 4.2 to 340 ng L^−1^ ^27^; clearly, the MND contents of oxidized red wines are higher than non‐oxidized ones, and therefore a variation of 1 to 21 times in the perception threshold is recorded (16 ng L^−1^). MND content was analyzed not only in wines of different origins and vintages, but also in those coming from different winemaking processes, including wines produced with white grapes harvested at maturity (dry white wine), at the overripe stage (sweet overripe wines) botrytized (botrytized white wine), and rosé and sweet fortified wines.

Wines were classified based on the intensity of their oxidative flavours. For non‐oxidized wines, MND concentration changed from one wine to another, the lowest concentrations being found in dry white wines (Table 3). Non‐oxidized botrytised wines reached maximum levels at 60.1 ng L^−1^. For all wines analyzed, the MND concentration was mostly below or close to the perception threshold (16 ng L^−1^ for model dry wines, 97 ng L^−1^ for sweet wines). Fortified sweet wines do not appear on this list because they are considered systematically oxidized. By contrast, MND content of all oxidized wines (except white wines) was systematically higher, reaching 293.8 ng L^−1^ for oxidized botrytised white wine.

**Table 3:** MND content in oxidized (Ox) and not‐oxidized (N‐Ox) samples

	Not oxidized N‐Ox (ng L^−1^)	Oxidized Ox (ng L^−1^)
	Min‐max	Min‐max
Red***	1.9–42.4	22.3–330.1
White	2.9–9.2	8.7–23.1
Overripe white***	2.8–14.0	17.9–65.2
Botrytised white***	7.7–60.1	40.6–293.8
Rosé***	2.8–10.5	51.6–103.6
Fortified red***		60.2–191.3

*Note*: Samples taken from 120 different wines. Adapted with permission from Pons *et al*.^27^ Copyright © 2013 American Chemical Society.

*** Significantly different at *P* < 0.001.

Wines from warm climate areas, such as southern Apulia (Italy) or Rioja (Spain), were reported to contain relatively high amounts of MND, with a maximum of 467 ng L^−1^.^53^, ^54^ We hypothesize an increased synthesis of fatty acid precursors in grapes ripening at higher temperatures, leading to either higher levels of MND or earlier reached maxima. However, this hypothesis should be confirmed in future research.

In wines obtained from Verdicchio grapes, a white grape variety, mainly grown in the Marche region (Italy), the presence of MND seems capable to impart a positive and characteristic note to the product.^55^ To understand the contribution of this compound in the Verdicchio's wines aroma, MND was added to a ‘neutral’ Verdicchio. Panellists observed that the fruity note was masked only by the addition of high quantities of MND equal to 500 ng L^−1^ because the anise note predominated in this wine. The samples preferred by panellists were those with an MND concentration between 25 and 50 ng L^−1^ and no oxidative notes were detected in the analyzed samples.^55^

### Role of aging, oxygen and sulphur dioxide in the formation of MND

Unexpectedly, no relationship between age and MND level was found in a sample set of 67 red wines.^27^ It follows that high levels of MND can be found not only in mature wines, but also in younger ones (< 5 years) and that precursors contribute to determining the aroma aging potential of red wine.

MND and MND‐related hydroxyketones (Fig. 4) were analyzed in a set of 167 wines ranging in age from 1 to 28 years.^51^ Hydroxyketones and MND were positively correlated with the wine age. An oxidation experiment of these MND‐related compounds in red wine confirmed that one or both ketols are precursors to MND. Therefore, aging results in an increase of these compounds and particularly MND. The rate of increase of MND can be also considered a discriminant between wines capable of aging for a long time, keeping young flavours and wines more prone to oxidative decline that quickly express oxidative nuances, as occurs mostly with wines exposed to oxygen during the winemaking process. Our research group recently observed relevant increases of MND in a Negroamaro wine after aging in different vessels, including glass bottle, amphora and five types of oak barrels. The final levels of MND were in the range 0.081–0.467 μg L^−1^ compared to 0.016 μg L^−1^ before aging.^54^ Interestingly, Mislata *et al*.^53^ reported even 11‐fold increases on MND in Rioja red wines after induced oxidation, reaching concentrations above 1 μg L^−1^.

**Figure 5:**
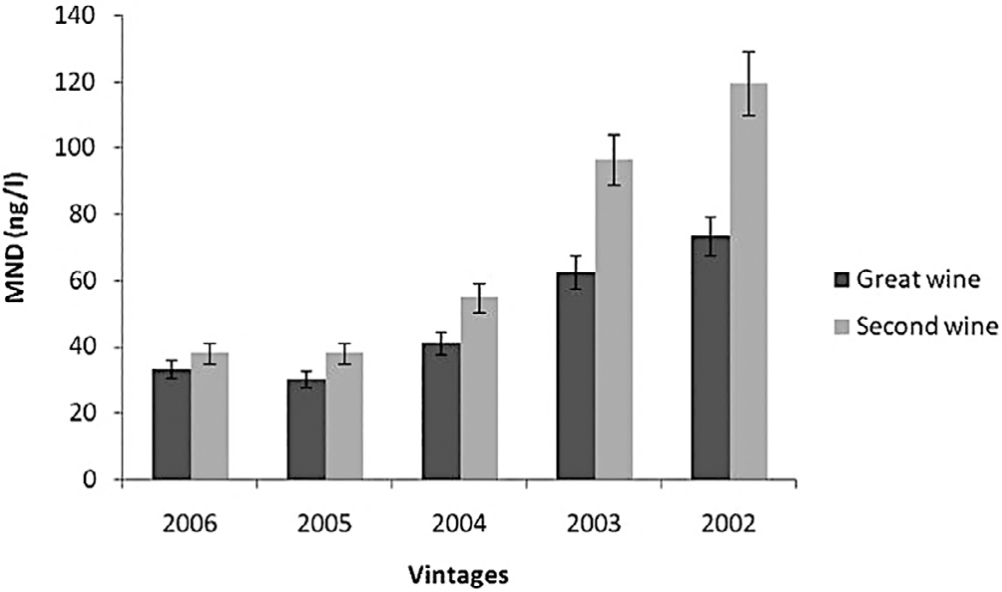
Comparison of levels of MND found in a great and a modest wine from the same producer for several years.^27^ Copyright 2013 American Chemical Society.

Pons *et al*.^27^ carried out the quantification of MND content in two red wines corresponding to the first and second type of wine from the same producer and for 5 years (Fig. 5). It was observed that, during the early phase of aging, MND concentrations in both wines were below the perception threshold and were similar. After 5 years of bottle aging, the MND concentration had increased to 73 ng L^−1^ in the first wine and 120 ng L^−1^ in the second.^27^ Analogously, 13 red wines of different vintages (1982–2000) from the same winery where analyzed in 2008 and showed levels ranging from a small amount up to 330 ng L^−1^, with the highes levels found in the oldest wines.^56^ It has previously been shown that the MND formation in red wines is associated with an oxidative mechanism: consequently, the higher concentration of MND in the second type of wine could be the result of a lower antioxidant shield, comprising a lower capacity to undergo oxidation keeping wine's flavour.

Therefore, oxygen availability is a crucial parameter during bottle aging as regards the onset of premature aging: it depends on the oxygen dissolved at bottling and on the level of closure's permeability.^27^ Addition of oxygen determined a steeper increase in MND concentration compared to control wines. At the end of the experiment, the level of MND had reached 80 ng L^−1^, which was higher than its perception threshold. Therefore, the role of oxygen and oxidation mechanisms on MND formation in red wines appears to be important. A recent paper by the same research group^57^ evaluated the effect of different closures, with differing oxygen transmission rates (OTR), on the levels of MND over 10 years of aging. An increase of MND was resported, even though this was not linear over time, with a maximum reached after 66 months and a subsequent plateau stage. Its evolution was significantly correlated with the SO_2_ loss. An increase of OTR appeared to be related to increases in MND, but, more generally, MND levels were affected by the combination of several parameters involved in wine aging, such as oxygen availability, SO_2_ depletion and wine aging potential.

An unexpected fate of MND under oxidative conditions was observed during accelerated oxygenation of fortified (mystelle‐type) sweet wine in transparent glass jars.^58^ A decrease of MND was observed after oxygenation and this effect was attributed to photo‐oxidation of MND in 3‐hydroxy‐3‐methyl‐2,4‐nonanedione (HMND), as previously described.^52^

The evaluation of the direct activity of SO_2_ towards MND showed that, at high SO_2_ levels, there was a slight decrease (10%) in MND concentration (Table 4).

**Table 4:** Sulphur dioxide effect on the amount of MND determined in model wine solution after 7 days (*n* = 2)

	SO_2_ (mg L^−1^)					
	0	10	20	30	40	60
MND (ng L^−1^)	100 (5)	100 (7)	98 (7)	99 (5)	97 (8)	85 (7)

*Note*: Adapted with permission from Pons *et al*.^27^ Copyright © 2013 American Chemical Society.

Therefore, the reactivity of SO_2_ with this diketone is low compared to others monocarbonyls such as acetaldehyde, formaldehyde or diketones such as diacetyl.^27^, ^59^ Sulphur dioxide, therefore, at the levels found in red wines, is not able to effectively bind MND and modify its concentration. Instead, its effectiveness is related to the prevention of the oxidation phenomena leading to MND formation.

## OTHER MOLECULAR MARKERS OF COOKED FRUIT AROMA

### γ‐Nonalactone

Lactones are cyclic esters of hydroxy‐fatty acids.^60^ They can arise from several origins, including fermentation, oak ageing and oxidative ageing. However, glycosidic precursors are also found in grapes and grape dehydration can further contribute to their presence in wines.^60^, ^61^, ^62^, ^63^ γ‐nonalactone has been identified in many wines.^60^, ^64^, ^65^ Concentrations are higher in vintage red wines^66^, ^67^ and in red wines aged in oak barrels.^68^ This compound is related to coconut and cooked peaches nuances, as well as being associated with wilting of the berries, which often occurs when the grapes are overripe or when the berries are infected with *Uncinula necator*.^69^

**Figure 6:**
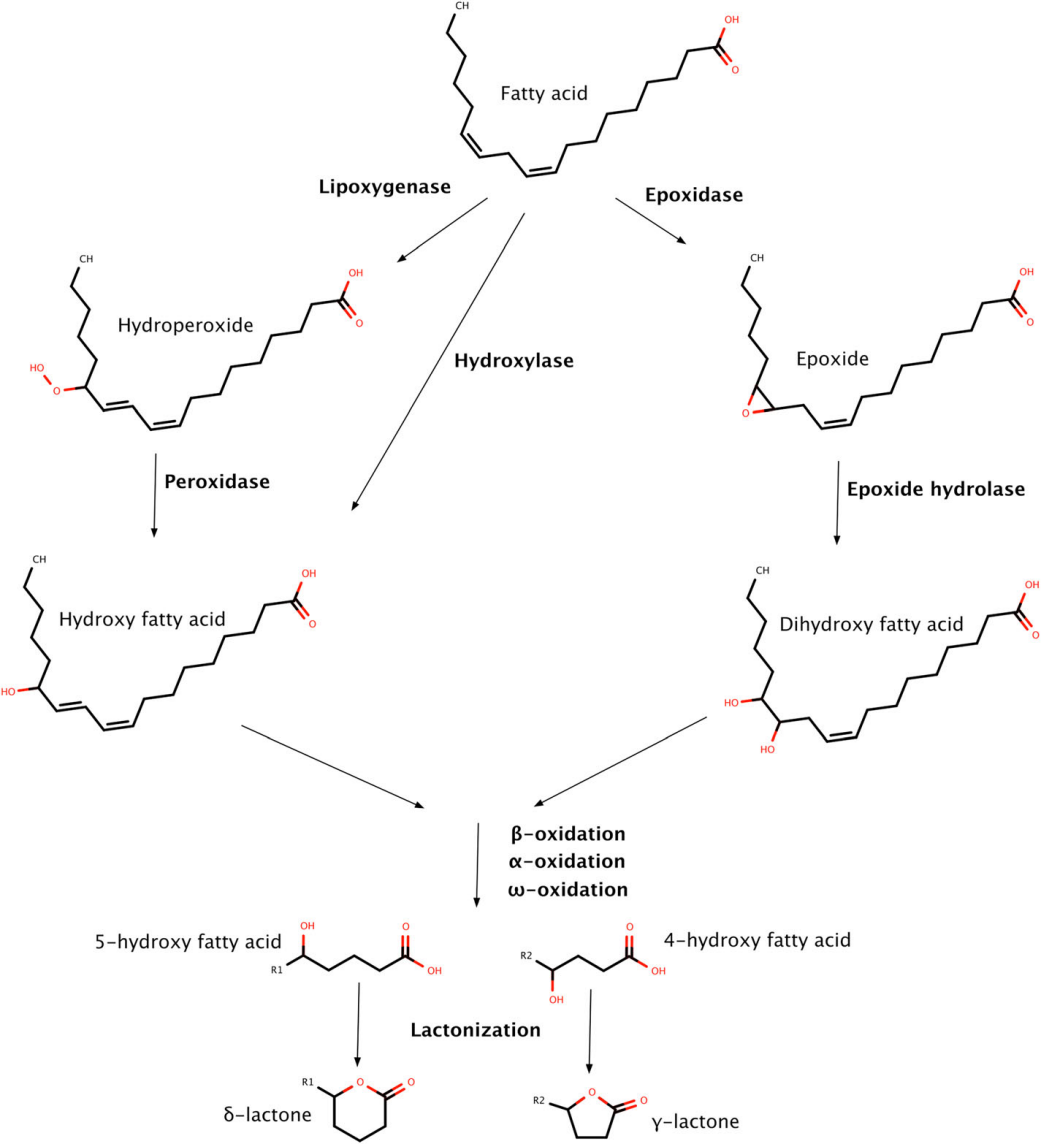
Biosynthetic pathways of lactones in fruits.^70^, ^71^, ^73^

Lactones biosynthesis has been partially elucidated in some fruits (i.e. strawberry, peach, mango) and has been definitely related to fatty acid metabolism.^70^, ^71^ A key step of lactones biosynthesis is fatty acid hydroxylation, an oxidative step catalyzed either by hydroxylases or by the successive activity of epoxidases and epoxide hydrolases, as well as lipoxygenases followed by peroxidases (Fig. 6). Further oxidation of the hydroxy fatty acids (either β‐ or α‐ or ω‐oxidation) would produce 4‐ or 5‐hydroxyacids, whose cyclization (or lactonization) could be either spontaneous or catalyzed by enzymes.^71^ In this regard, a gene was identified in peaches encoding for a novel type of alcohol acyl transferase (ID: PPN001H09) that could be a candidate for this step.^72^

**Figure 7:**
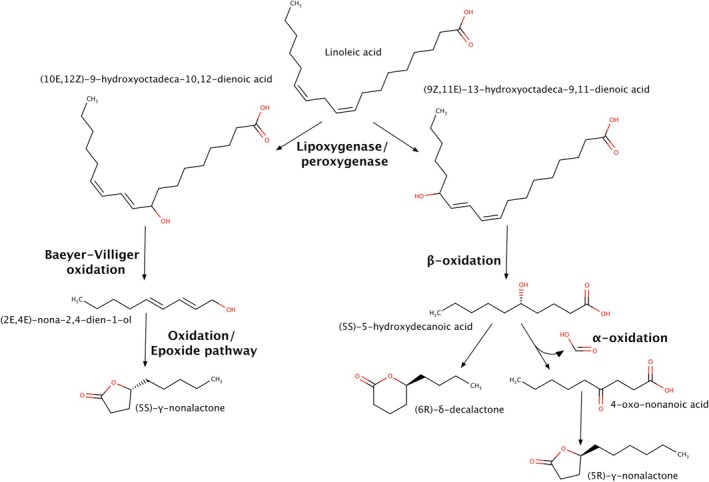
Suggested formation pathways of γ‐nonalactone.^60^, ^73^, ^74^

γ‐nonalactone has been identified as oxidation product of linoleic acid^73^ in wine,^74^ as well as in other beverages and food products, such as beer^75^ and butter.^76^ Its formation involves the presence of precursors in must, such as C‐18 hydroxyacids from lipoxygenase/peroxidase activities catalysing oxidation of linoleic acid, as well as their cleavage products such as 4‐oxo‐nonanoic acid (Fig. 7).

These precursors are then bio‐transformed in γ‐nonalactone by yeasts.^73^, ^74^ Recently, 4‐oxonanoic acid, which is a direct precursor of β‐nonalactone, has been detected in the must.^74^ The biotransformation of this hydroxy acid into γ‐nonalactone by *S. cerevisiae* was reported with a yield of approximately 95% and maximum measured concentrations of 60 μg L^−1^ and 28.7 μg L^−1^ in the Merlot and Cabernet Sauvignon musts, respectively.^74^ The involvement of fungal metabolism in the formation of γ‐nonalactone was also suggested by Schueuermann *et al*.,^77^ who found significantly higher levels of this compound in juices obtained from bunches infected by *Penicillium expansum* compared to juices from healthy grapes as well as from grapes infected with other fungal pathogens. Analogously, Barata *et al*.^78^ reported a three‐fold increase of γ‐nonalactone in red wines from sour rot grapes compared to control wines.

Using GC‐olfactometry, Pons *et al*.^2^ analyzed the aroma extracts of prematurely aged red wines and reported, for one of the aroma‐active regions, an overripe peach descriptor, corresponding to the elution peak of γ‐nonalactone.

### Massoia lactone

**Figure 8:**
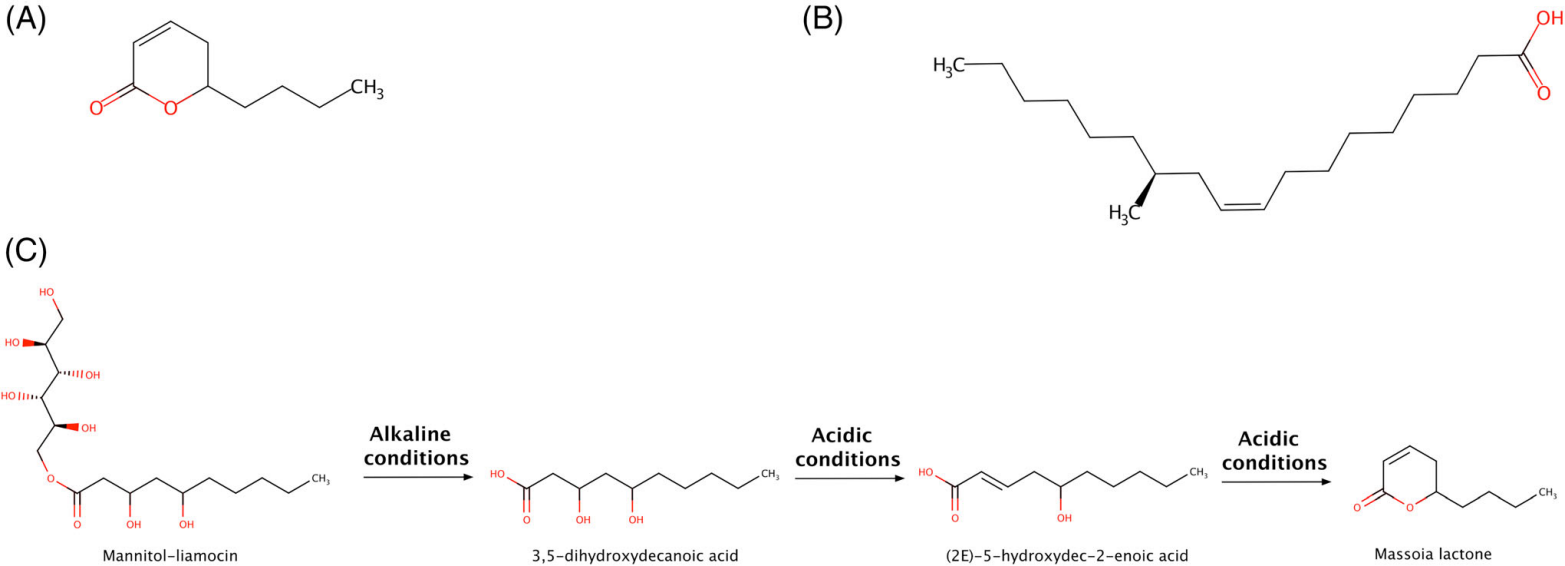
Structure of massoia lactone (A) and ricinoleic acid (B). Biosynthetic pathway of massoia lactone by *Aureobasidium spp*.^81^, ^82^

Massoia lactones are 10, 12 and 14 carbon chain α‐β unsaturated lactones. The C‐10 massoia lactone (Fig. 8A) is the main constituent of the *Massoia aromatica* (Becc) essential oil, a tree form Indonesia.^79^ The C‐10 massoia lactone has also been found in musts and wines, even though scarce information is available about its formation.^80^

Some studies suggest ricinoleic acid (12‐hydroxy‐9‐cis‐octadecenoic acid) (Fig. 8B) as precursor of massoia lactone.^80^ Ricinoleic acid is the main constituent of castor (*Ricinus communis* L.) oil^83^ but it is not commonly found in other plants. Ricinoleic acid can be considered the hydroxylation product of oleic acid. Therefore, this could be a step in biosynthesis of massoia lactone according to the pathway described in Fig. 8. Pons *et al*.^80^ suggest that massoia lactone would form in a similar way, as also supported by the findings of Dupé *et al*.,^84^ who reported massoia lactone as a product of harsh degradation of methyl‐ricinoleate. The hypothetical involvement of a desaturase could be considered. In this regard, the involvement of a bifunctional desaturase/hydroxylase has been reported for the biosynthesis of γ‐decalactone in strawberry.^85^ Another pathway suggested by Pons *et al*.^80^ involves the cleavage of hydroperoxides via hydroperoxide lyases, although Deshpande *et al*.^70^ consider unfavorable this step for lactone biosynthesis.

It has also been reported to be released by liamocins, antifungal metabolites produced by fungi belonging to the *Aureobasidium* genus^86^, ^87^ (Fig. 8C).

Given the widespread diffusion and abundance of *Aureobasidium spp*. on grapes,^81^ the contribution of this fungus to the presence of massoia lactone in musts and wine should be evaluated, as suggested by the literature about the potential contribution of endophytic microrganisms to wine aroma.^88^ Massoia lactone itself showed a strong antifungal activity.^87^

Chou *et al*.^89^ included this compound among those characterizing wines from shriveled Shiraz grapes. An interesting method to assess grape aroma potential was recently applied to Tempranillo.^90^ The phenolic and aromatic fractions obtained from grapes, and aged in strict anoxia 7 weeks at 45 °C or 24 h at 75 °C, developed strong aroma. The odorants identified with GC‐O after incubation at 75 °C included massoia lactone, associated with coconut nuances. This would support the hypothesis of massoia lactone precursors in grape and their subsequent biotransformation. The analysis of Cabernet Sauvignon and Merlot musts and wines characterized by dried fruit and cooked fruit aromas enlightened the role played by massoia lactone, associated with coconut and fig descriptors.^80^ Levels up to 68 μg L^−1^ and 20 μg L^−1^ were found in musts and wines characterized by these descriptors, respectively. The relation between this compound and the dried fruit nuances was clear in musts, whereas it was not clear in young and old red wines. The reduction of massoia lactone to δ‐decalactone by *S. cerevisiae* was also reported. Finally, a link between growing temperature and the levels of massoia lactone in musts was suggested.^80^ Pons *et al*. carried out a vertical evaluation of massoia lactone in a red wine over several vintages from two estates. The highest concentration (16 μg L^−1^), higher than the detection threshold, was found for the 2003 vintage, characterized by high summer temperatures.^80^ This would suggest that global warming's outcomes could include increased occurrence of cooked fruit aromas in wines. A recent study confirmed that the levels of massoia lactone in Cabernet Sauvignon and Merlot musts and wines increased because harvesting was delayed and cooked fruit aroma was more intense. However, the levels of massoia lactone showed no linear correlation with the intensity of the cooked fruit aroma, whereas other compounds showed significant correlations.^6^

### (*Z*)‐1,5‐octadien‐3‐one

**Figure 9:**
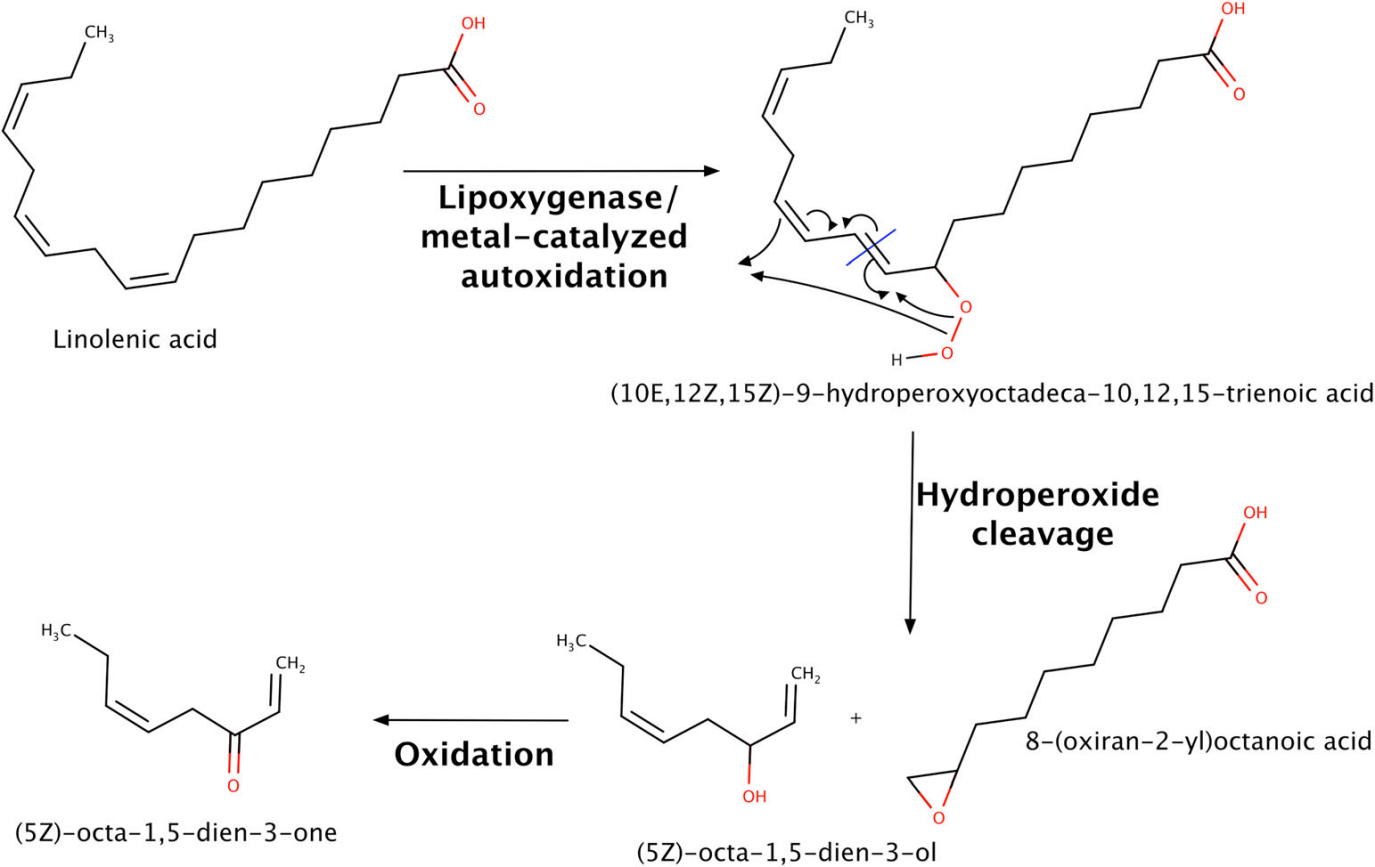
Formation pathway of (*Z*)‐1,5‐octadien‐3‐one.^91^, ^93^, ^94^

(*Z*)‐1,5‐octadien‐3‐one is reported as a product of fatty acid oxidation, and has geranium‐like and metallic odor attributes.^91^ In grapes, its presence has been demonstrated through GC‐O, and it is associated with the development of pathogens such as *U. necator*, also called powdery mildew.^92^ The compound can be generated by the oxidation of precursors such as α‐linolenic acid,^91^, ^93^, ^94^ in the presence of metal ions or lipoxygenase (Fig. 9). Hydroperoxide cleavage would lead to the formation of (*Z*)‐1,5‐octadien‐3‐ol, further oxidized to (*Z*)‐1,5‐octadien‐3‐one. However, further investigations are needed to confirm the formation pathway of (*Z*)‐1,5‐octadien‐3‐one in grapes and wines.

Concentrations of (*Z*)‐1,5‐octadien‐3‐one in musts characterized by dried fruit aromas reached 90 ng L^−1^, thus exceeding the detection threshold of this compound (9 ng L^−1^).^93^ Furthermore, Allamy *et al*.^93^ added increasing amounts of this compound to a Merlot must. They reported it to contribute to the nuance of dried fig at concentrations between 64 and 96 ng L^−1^. Above that level, there was an onset of geranium leaf attributes. Sulphur dioxide was reported to decrease (*Z*)‐1,5‐octadien‐3‐one levels through sulfonate adducts formation; the addition of 30 mg L^−1^ of SO_2_ led to a 60% reduction of (*Z*)‐1,5‐octadien‐3‐one in a model must and an 81% reduction in a model wine.^93^ Moreover, Darriet *et al*.^92^ found that *S. cerevisiae* enzymatic activities could reduce (*Z*)‐1,5‐octadien‐3‐one to the less potent odorant (*Z*)‐5‐octen‐3‐one. In a study paper, Allamy *et al*.^6^ reported increasing concentrations of this ketone in late‐harvested Cabernet Sauvignon and Merlot compared to early harvest, contributing to the cooked‐fruit aroma. The increases were approximately five‐ and seven‐fold over 18 days of ripening/over‐ripening in Merlot and Cabernet Sauvignon musts, respectively. However, this compound was not found in wines, probably because of its reduction by yeasts.^6^

### (2*E* ,4*E* ,6*Z* )‐nonatrienal and *trans*‐4,5‐epoxy‐(*E*)‐2‐decenal

**Figure 10:**
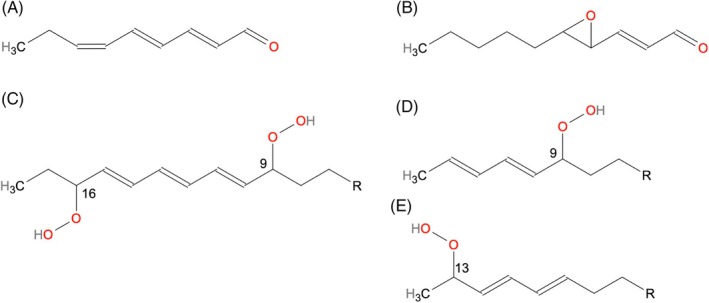
Structures of (2*E*,4*E*,6*Z*)‐nonatrienal (A), *trans*‐4,5‐epoxy‐(E)‐2‐decenal (B), 9,16‐dihydroperoxy‐10,12,14‐octadecatrienoate of α‐linolenic acid (C), 9‐ hydroperoxide of linoleic acid (D) and 13‐hydroperoxide of linoleic acid (E).

The polyunsaturated aldehyde (2*E*,4*E*,6*Z*)‐nonatrienal (Fig. 10A) is known as an oxidation product of α‐linolenic acid, reported as a key odorant in several food matrices (e.g. blended dry beans, oat flakes, black tea), with an oat flake‐like nuance and very low odour threshold.^95^, ^96^, ^97^ Buttery,^96^ who first identified this compound, indicated that the formation of an aldehyde with three unsaturated bonds is difficult to be explained as the result of cleavage of a monohydroperoxide. Nevertheless, Schuh and Schieberle^95^ induced its formation during the autoxidation of linolenic acid. It was suggested that (2*E*,4*E*,6*Z*)‐nonatrienal could derive from the cleavage of a dihydroperoxide (9,16‐dihydroperoxy‐10,12,14‐octadecatrienoate) of α‐linolenic acid (Fig. 10C), which can arise as a consequence of photo oxidation or enzymatic oxidation, as previously shown by Neff *et al*.^98^ and Grechkin *et al*.^99^, respectively.

*Trans*‐4,5‐epoxy‐(*E*)‐2‐decenal (Fig. 10B) is an unsaturated epoxy‐aldehyde reported as a product of the cleavage of 9‐ and 13‐ hydroperoxides of linoleic acid (Fig. 10D,E).^100^ Its odour threshold is very low and its occurrence in foods as a potent odorant is widely reported (e.g. soybean oil, stored beef, wheat bread crumb, popcorn, roasted sesame seeds, dried hop cones).^100^, ^101^, ^102^

Recent research reported both aldehydes in toasted oak wood, and characterized their aroma impact by GC‐ olfactometry, indicating metal and puff pastry nuances for (2*E*,4*E*,6*Z*)‐nonatrienal and *trans*‐4,5‐epoxy‐(*E*)‐2‐decenal, respectively.^103^ A subsequent investigation reported, for the first time, both aldehydes in wines and spirits.^104^ Their olfactory detection thresholds were determined, corresponding to 16 ng L^−1^ and 60 ng L^−1^ for (2*E*,4*E*,6*Z*)‐nonatrienal and *trans*‐4,5‐epoxy‐(*E*)‐2‐decenal, respectively. The analysis of 66 wine samples highlighted a maximum amount of (2*E*,4*E*,6*Z*)‐nonatrienal (441.3 ng L^−1^) in a red wine and a maximum amount of *trans*‐4,5‐epoxy‐(*E*)‐2‐decenal (524.4 ng L^−1^) in a white wine. Despite their potentially high reactivity, as a result of both the carbonyl functional group and the unsaturated bonds, both aldehydes could be detected in non‐negligible amounts. Toasted oak wood could also release large amounts of (2*E*,4*E*,6*Z*)‐nonatrienal in red wine, whereas *trans*‐4,5‐epoxy‐(*E*)‐2‐decenal appeared to give non‐volatile hydroxysulfonate adducts with SO_2_. However, when comparing oak‐aged red wines with wines aged in steel vats, no significant difference was observed in the levels of both aldehydes. It was suggested that toasted oak wood is not the only source of these potent odorants and that their formation could occur from precursors in grapes. Additionally, the impact of oak wood maturation on the levels of α‐linolenic and linoleic acid was analyzed. The former was not detected in any time, whereas the latter was released by wood and decreased during aging. Correspondingly, an increase of the aldehydes was observed, supporting the hypothesis of the role of fatty acids as precursors in wine.^104^ Finally, the sensory impact of these aldehydes was assessed by supplementing a red wine with increasing amounts of the odorants. The observed effect was a decrease of fresh fruity notes and an increase of cooked/dried fruit notes, even at concentrations below their sensory threshold, via a perceptual interaction mechanism.^104^

## IMPACT OF THE GRAPE HARVEST DATE AND OVER‐RIPENING ON MOLECULAR MARKERS RESPONSIBLE FOR THE COOKED FRUIT AROMA

Grape exposure at high temperatures during ripening clearly affects the development of the fruity aroma, favouring the onset of cooked fruit and oxidized prune nuances, with a potential impact on the terroir expression.^105^ Merlot and Cabernet Sauvignon grapes were submitted to off‐vine drying process to assess the effects on the dried fruits aroma in musts and wines.^5^ Higher levels of MND and (*Z*)‐1,5‐octadien‐3‐one were reported in wines with dried fruit notes. As regards lactones, γ‐decalactone and massoia lactone were found at higher levels in musts with dried fruit profiles, whereas γ‐nonalactone was more abundant in wine with dried fruit aroma. Furaneol and homofuraneol were also reported as contributing to dried fruit aromas. A reconstitution experiment confirmed the synergic effect of these compounds in determining the dried fruit aroma. Moreover, the effect of light with respect to increasing compounds responsible for these nuances was confirmed. It was suggested that drying conditions would enhance *in situ* oxidation phenomena involving lipids via either increased lipoxygenase activity or increased ROS generation. Both causes have been previously documented in literature. Costantini *et al*.^106^ highlighted increased lipoxygenase activity during Malvasia berry dehydration. In particular, lipoxygenase activity increased by almost 60% in correspondence of a weight loss of 11.7%. On the other hand, high light and temperature stress in plants has been reported causing increased levels of ROS.^107^

A recent study by Allamy *et al*.^6^ evaluated how the fine tuning of the grape harvest date could allow modulation of the aroma components in musts and wines and the equilibrium among herbaceous, fresh fruit and cooked fruit nuances. The basis of these changes is a result of the increased impact of some aroma compounds in the must and in the wine, such as furaneol, MND, (*Z*)‐1,5‐octadien‐3‐one, massoia lactone and γ‐nonalactone (Table 5). For Merlot and Cabernet Sauvignon, a delay of harvest date in the range 4–12 days was resported, according to vintage and cultivar, with an increase in the intensity of the cooked fruit aroma. A delay in the grape harvest increased the concentration of lactones, furanones, and above all MND and (*Z*)‐1,5‐octadien‐3‐one concentrations. Most of the compounds involved in the perception of overripe grapes cooked fruit aroma were detected at higher levels in wines produced from late harvest dates. It is clear that aroma compounds, such as furans, lactones and ketones, are associated, through perceptual interaction, with the development of cooked fruit nuances in the wine.

**Table 5:** Impact of the harvest date (D)^a^ on the composition of volatile compounds of Merlot and Cabernet‐sauvignon musts and wines (2014 experiment)

Compound	Sample	Merlot					Cabernet‐Sauvignon				
		D‐6	D	D+4	D+12	*P*	D‐3	D	D+2	D+7	*P‐*value
MDN (1)	Must	41.9 ± 4.4	85.6 ± 2.2	42.3 ± 5.1	38.3 ± 1.2	***	35.0 ± 2.9	50.2 ± 2.6	49.1 ± 3.8	38.2 ± 2.2	*
	Wine	20.7 ± 1.4	29.7 ± 1.9	34.7 ± 1.7	56.0 ± 1.6	*	27.1 ± 1.9	30.1 ± 2.6	27.7 ± 1.2	35.5 ± 1.9	*
γ‐Nonalactone (2)	Must	2.9 ± 0.8	3.0 ± 0.4	3.2 ± 0.5	3.1 ± 0.9	NS	Tr	Tr	1.3 ± 0.6	1.5 ± 0.3	*
	Wine	6.3 ± 0.4	6.4 ± 0.5	8.2 ± 0.9	24.9 ± 1.9	***	9.9 ± 2.3	8.8 0.3	8.9 ± 0.4	19.4 ± 0.9	***
Massoia lactone (2)	Must	8.2 ± 0.3	6.4 ± 0.6	7.3 ± 0.8	9.0 ± 0.9	*	4.9 ± 0.3	5.0 ± 0.2	4.8 ± 0.4	4.5 ± 0.3	NS
	Wine	3.7 ± 0.6	18.6 ± 1.8	22.2 ± 1.1	26.8 ± 1.9	***	4.5 ± 0.3	4.6 ± 0.4	19.9 ± 1.4	19.7 ± 1.7	***
Furaneol (2)	Must	9.2 ± 0.5	8.7 ± 0.6	8.6 ± 0.9	9.3 ± 0.5	NS	8.1 ± 0.5	8.0 ± 0.6	8.2 ± 0.3	8.2 ± 0.5	NS
	Wine	36.0 ± 2.9	43.3 ± 4.4	49.8 ± 4.8	62.8 ± 2.4	**	41.1 ± 2.5	42.7 ± 2.5	42.2 ± 2.9	68.7 ± 3.1	**
Homofuraneol (2)	Must	4.0 ± 1.6	2.9 ± 0.8	5.0 ± 1.2	7.6 ± 0.4	*	2.2 ± 0.1	2.3 ± 0.1	2.4 ± 0.1	2.4 ± 0.2	NS
	Wine	5.6 ± 1.0	5.0 ± 0.9	4.6 ± 1.1	5.1 ± 1.1	NS	4.9 ± 0.4	5.3 ± 0.3	4.1 ± 0.5	5.2 ± 0.4	NS
(*Z*)‐1,5‐Octadien‐3‐one (1)	Must	54.5 ± 3.2	150.5 ± 9.1	252.4 ± 5.8	256.0 ± 8.1	***	7.0 ± 2.6	44.1 ± 2.8	45.2 ± 3.3	50.7 ± 2.5	***
	Wine	Tr	Tr	Tr	Tr		Tr	Tr	Tr	Tr	

*Note*: Concentrations expressed in (1) ng L^*−*1^ and (2) μg L^*−*1^. NS, not significant; Tr, trace. Adapted with permission from Allamy *et al*.^6^ (CC BY 4.0).

^a^ D, harvest at technological maturity; D‐6: harvest 6 days before technological maturity; D+4: harvest 4 days after technological maturity; D+12: harvest 12 days after technological maturity.

## IMPACT OF *PLASMOPARA VITICOLA* ON THE DEVELOPMENT OF COOKED FRUIT AROMAS

**Figure 11:**
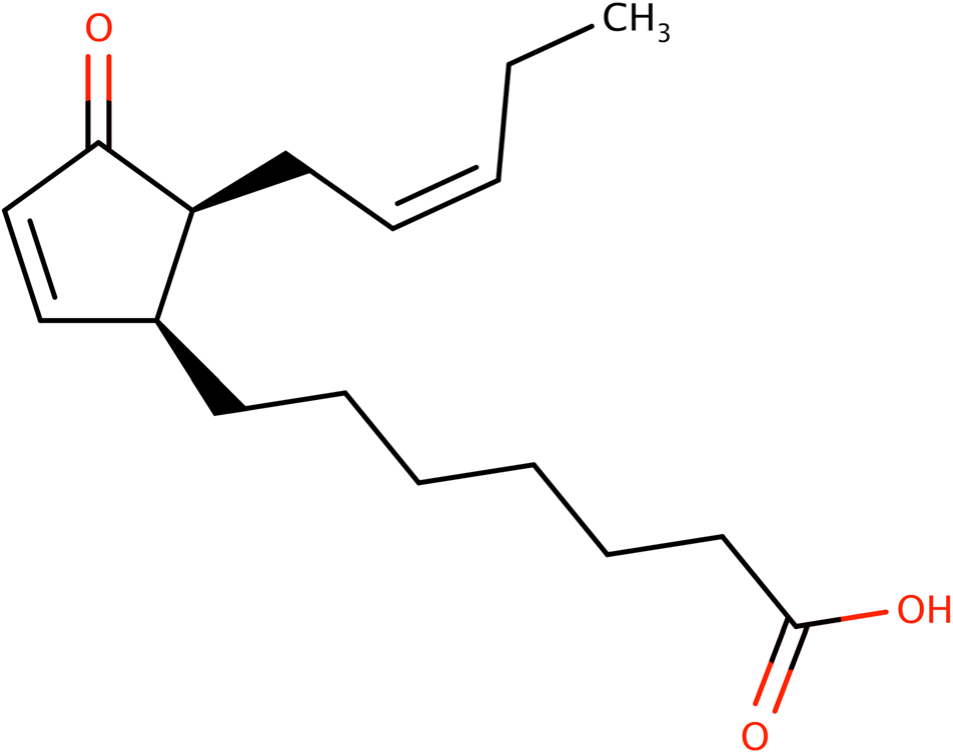
Structure of *cis*‐(+)‐12‐oxophytodienoic acid.

Berries infections by pathogens such as *P. viticola*, known as downy mildew, modify the grape's aromatic profile. These changes were characterized as increase of green and herbaceous notes in wines, related to figs and cooked fruit nuances.^69^ GC‐olfactometry highlighted the association of such aroma descriptors with an increase of the compounds deriving from fatty acid oxidation: lactones, such as γ‐octalactone, γ‐nonalactone and γ‐ decalactone, (*Z*)‐1,5‐heptadien‐3‐one and (*Z*)‐1,5‐octadien‐3‐one, and MND. MND was the most abundant in wines produced from grapes that include diseased berries. Finally, Merlot wines produced with infected berries by *P. viticola* presented a greater intensity of the ‘cooked fruit’ character and sometimes green nuances. It should be considered that lipid signalling has been highlighted as a response to *P. viticola* infections, involving changes in linolenic acid contents and lipid peroxidation.^108^ This metabolic change has partly been related to the biosynthesis of jasmonates, involved in the stress response.^109^, ^110^ Interestingly, the biosynthesis mechanism proposed for jasmonates^110^ involves the peroxidation of linolenic acid, as well as the formation of the intermediate *cis*‐(+)‐12‐oxophytodienoic acid (Fig. 11), with a cyclopentenone moiety. We hypothesize that this pathway and its intermediates could provide other candidate precursors for MND and other lipid‐derived volatile compounds. Other studies highlighted that *P. viticola* infection leads to changes in the lipidomic profile of grapes, with the appearance of unusual fatty acids,^111^ such as eicosapentaenoic acid and arachidonic acid, as well as the accumulation of ROS.^108^, ^112^ This could contribute to the increase of lipid oxidation related volatile compounds, including those associated to cooked fruit aroma.

## BRIDGING GRAPE LIPIDOMICS AND WINE FLAVOROMICS

Table 6 reports an overview of the fatty acid‐derived volatile compounds examined in this review, as related to cooked fruit aroma and premature aging in red wines.

**Table 6:** Overview of the fatty acid‐derived volatile compounds examined in this review, as related to cooked fruit aroma and premature aging in red wines

Compound	Candidate precursors	Main findings	References
3‐methyl‐2,4‐nonanedione	Furan fatty acids: 10,13‐epoxy‐11,12‐dimethyloctadeca‐10,12‐dienoic acid and 12,15‐epoxy‐13,14‐dimethyleicosa‐12,14‐dienoic acid Hydroxyketones α‐linolenic acid via *cis*‐(+)‐12‐oxophytodienoic acid	Loss of fresh fruity flavours, onset of plum and fig nuances Concentration‐dependent sensory impact The most potent key food odorant for OR1A1's receptor Increases during aging as a result of oxidation/photooxidation Its levels are affected by precursors, oxygen availability, sulphur dioxide Higher levels in wines from grapes submitted to off‐vine drying Delayed harvest increases its levels in wine Increases together with cooked fruit notes in wines form berries affected by *Plasmopara viticola*	1, 2, 5, 27, 28, 36, 46, 47, 51, 52, 53, 54, 59, 69
γ‐nonalactone	Linoleic acid via C‐18 hydroxyacids 4‐oxononanoic acid	Coconut and cooked peaches nuances Reported at higher levels in wines with dried fruit aroma Synergic effect in determining dried fruit aroma Its formation involves both enzymatic and autoxidation steps Possible enhancement of formation due to fungal metabolism Delayed harvest increases its levels in wine Increases together with cooked fruit notes in wines form berries affected by *P. viticola*	5, 69, 70, 71, 73, 74, 77, 78
Massoia lactone	Ricinoleic acid Oleic acid Liamocins	Coconut and fig nuances, with a role in dried fruit and cooked fruit aroma Reported at higher levels in musts with dried fruit aroma Synergic effect in determining dried fruit aroma Possible role of endophytic microrganisms in its formation Increases with increasing ripening and harvesting temperatures Delayed harvest increases its levels in wine	5, 6, 80, 88
(*Z*)‐1,5‐octadien‐3‐one	α‐linolenic acid	Geranium‐like and metallic attributes. Contributes to dried fuit and fig aroma Associated to pathogens such as *Unicinula necator* Forms sulfonate adduction products with sulphur dioxide Reduced by *Saccharomyces cerevisiae* Increases during ripening and over‐ripening. Higher levels in wines from grapes submitted to off‐vine drying Increases together with cooked fruit notes in wines form berries affected by *P. viticola*	6, 91, 92, 93, 94
(2*E*,4*E*,6*Z*)‐nonatrienal	α‐linolenic acid via formation of (9,16‐dihydroperoxy‐10,12,14‐octadecatrienoate)	Metal nuances Decreases fresh fruity notes and increases cooked/dried fruit notes Released also from toasted oak wood Does not form adducts with sulphur dioxide	95, 103, 104
*Trans*‐4,5‐epoxy‐(*E*)‐2‐decenal	9‐ and 13‐hydroperoxides of linoleic acid	Puff‐pastry nuances Decreases fresh fruity notes and increases cooked/dried fruit notes Released also from toasted oak wood Forms adducts with sulphur dioxide	100, 103, 104

The lipid content in grape and wine is not negligible.^113^ Therefore, grape and wine lipidomics has been gaining increasing attention in recent years.^14^, ^23^ A review by Sherman and Pinu^114^ illustrated the applications of lipidomics in grape and wine‐related samples since 2015. The increasing knowledge regarding the lipid‐derived volatile compounds released by yeasts during fermentation and aging on lees was underlined. Also, unsaturated fatty acids have been studied as sources of C6 aldehydes and alcohols via peroxidation^115^ as well as of varietal thiols.^116^, ^117^ Overall, grape lipids are known to impact on wine aroma.^118^ However, in their review, Sherman and Pinu^114^ observed that the application of comprehensive lipidomics is still limited and suggested that special attention should be paid in future to lipidomics in relation to flavoromics development in wine, as already occurring in other food‐related applications.^7^, ^9^, ^114^, ^119^, ^120^ Indeed, the present overview indicates that grape lipidome not only plays a pivotal role in wine aroma development, but also could be strongly related to the impact of environmental variables on the development of grape‐derived flavours and off‐flavours. Lipidomics, as well as the chemistry of lipid oxidation, could therefore provide new insights on the effect of climate change on wine sensory quality.^121^

## CONCLUSIONS

In conclusion, this review reports the role of lipid‐derived volatile compounds in the onset of the Premox defect in red wines. Lactones and ketones deriving from fatty acid oxidation, in particular, play a crucial role in the appearance of cooked fruit, figs and plum nuances in prematurely aged red wines. Even though their formation pathways are not always clear, the biosynthesis of their precursors in grape, as well as their formation via enzymatic or radical reactions in grape, must or wine, has been highlighted in several studies. An understanding of the mechanisms underlying their formation and evolution in light of the principles of lipid chemistry could provide useful knowledge to adapt vineyard management (e.g. light exposure, disease management), harvest (e.g. date of harvest) and winemaking techniques (e.g. must enzymes management, control of oxidation) to the emerging issues related to climate change.

## AUTHOR CONTRIBUTIONS

IP, EO, GC, CD and GF were responsible for data curation. IP, EO and GF were responsible for writing the original draft. GC, CD and VMP were responsible for reviewing and editing. VMP was responsible for conceptualization, supervision and visualization.

## CONFLICTS OF INTEREST

The authors declare that they have no conflicts of interest.

## Data Availability

Data sharing is not applicable to this article as no new data were created or analyzed in this study.
